# Single‐nucleus RNA sequencing unveils critical regulators in various hippocampal neurons for anti‐N‐methyl‐D‐aspartate receptor encephalitis

**DOI:** 10.1111/bpa.13156

**Published:** 2023-03-21

**Authors:** Yunmeng Bai, Zhuhe Liu, Tinglin Qian, Yu Peng, Huan Ma, Hong Hu, Guangqing Cheng, Haixia Wen, Lulin Xie, Dong Zheng, Qingshan Geng, Jigang Wang, Honghao Wang

**Affiliations:** ^1^ Department of Nephrology, Shenzhen Key Laboratory of Kidney Diseases, Shenzhen People's Hospital, the First Affiliated Hospital, School of Medicine Southern University of Science and Technology Shenzhen China; ^2^ Department of Neurology, Guangzhou First People's Hospital, School of Medicine Southern China University of Technology Guangzhou China; ^3^ School of Traditional Chinese Medicine Southern Medical University Guangzhou China; ^4^ Department of Neurology, Nanfang Hospital Southern Medical University Guangzhou China; ^5^ Guangdong Cardiovascular Institute Guangdong Academy of Medical Sciences Guangzhou China; ^6^ Department of Neurology The Affiliated Brain Hospital of Guangzhou Medical University Guangzhou China

**Keywords:** anti‐N‐methyl‐D‐aspartate receptor encephalitis, hippocampus, neuronal types, single‐nucleus RNA sequencing

## Abstract

Anti‐N‐methyl‐D‐aspartate receptor (NMDAR) encephalitis is a neuropsychiatric disease with variable clinical manifestations caused by NMDAR autoantibody. The underlying molecular underpinnings of this disease are rarely characterized on a genomic scale. Anti‐NMDAR encephalitis mainly affects the hippocampus, however, its effect on gene expression in hippocampal neurons is unclear at present. Here, we construct the active and passive immunization mouse models of anti‐NMDAR encephalitis, and use single‐nucleus RNA sequencing to investigate the diverse expression profile of neuronal populations isolated from different hippocampal regions. Dramatic changes in cell proportions and differentially expressed genes were observed in excitatory neurons of the dentate gyrus (DG) subregion. In addition, we found that ATP metabolism and biosynthetic regulators related genes in excitatory neurons of DG subregion were significantly affected. *Kcnq1ot1* in inhibitory neurons and *Meg3* in interneurons also changed. Notably, the latter two molecules exhibited opposite changes in different models. Therefore, the above genes were used as potential targets for further research on the pathological process of anti‐NMDAR encephalitis. These data involve various hippocampal neurons, which delineate a framework for understanding the hippocampal neuronal circuit and the potential molecular mechanisms of anti‐NMDAR encephalitis.

## Graphical abstract

The authors investigate the diverse neuronal expression profile in different hippocampal regions of the anti‐NMDAR encephalitis mouse model, and dramatic changes in cell proportions and differentially expressed genes were observed, such as ATP metabolism and biosynthetic regulators related genes.

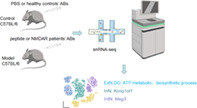

## 1 INTRODUCTION

Anti‐N‐methyl‐D‐aspartate receptor (NMDAR) encephalitis is a neuropsychiatric disease caused by the antibody‐mediated autoimmune response against NMDAR. The common clinical manifestations include psychiatric disturbances, cogniti impairments (executive functions and memory), epileptic seizures, and somnipathy [1, 2]. Although multiple researchers have reported treatment methods related to anti‐NMDAR encephalitis [2, 3], immunotherapy remains the pivotal clinical treatment contributing to a full recovery for patients at present [1]. However, even after receiving timely immunotherapy, about 15.9% of patients still experience one or more long‐term relapses, especially female patients [4]. Hence, it is urgent to explore the etiological therapy of anti‐NMDAR encephalitis.

In order to study the pathological process of anti‐NMDAR encephalitis, two experimental autoimmune encephalomyelitis (EAE) models in vivo have been successfully established so far, namely, the passive immunization model through an intracerebral transfer of purified human‐derived antibodies [5, 6], and the active immunization model via subcutaneous injection of a peptide from the amino‐terminal domain of the GluN1 subunit [7, 8]. In addition, in vitro cell models have also been established [2]. Previous research showed that brain sections immunostained with autoantibodies from patients with anti‐NMDAR encephalitis exhibited an intense reactivity with the neuropil in the hippocampus of rat brain [9]. Notably, in vitro and in vivo experiments show that the loss of NMDAR is evident in the hippocampus neurons [10]. This evidence indicated the susceptibility of hippocampus neurons to NMDAR autoantibodies. Despite that the pathogenicity of autoimmune antibodies has been proved [1, 11], the potential molecular mechanism of anti‐NMDAR encephalitis caused by the loss of NMDAR in hippocampal neurons remains largely unknown.

Generally, NMDARs mediate glutamatergic synaptic transmission and play a prominent role in synaptic plasticity, learning, and behavior [12]. Memory deficit is a common symptom occurred in both passive immunization and active immunization animal models of anti‐NMDAR encephalitis [5, 6, 7, 8]. Indeed, the formation of hippocampal memory depends on a canonical trisynaptic loop formed by the strong connection among its CA1, CA3, and dentate gyrus (DG) subfields, in which DG plays a key role [13]. Moreover, different neuron types participate in DG‐dependent mnemonic functions, particularly encoding, retrieval, and discrimination of similar memories [13]. With respect to the pathophysiology of anti‐NMDAR encephalitis, it is considered that synaptic long‐term potentiation (LTP) blocked by NMDAR autoantibodies results in the impairment of memory ability [14]. Recently, we used an amino‐terminal domain peptide from the GluN1 subunit (GluN1_356‐385_) against NMDARs to establish a new female C57BL/6 mouse active immunization model [7]. We found impaired LTP at Schaffer collateral‐CA1 synapses, which was consistent with previous reports about the CA1 and CA3 regions of the passive immunization model [15, 16]. In addition, the reduced NMDAR‐induced calcium influx is also observed [7]. Nevertheless, the key molecules involved in the aforementioned neuronal processes leading to memory deficit in anti‐NMDAR encephalitis still remain elusive.

In this study, by using single‐nucleus RNA sequencing (snRNA‐seq), we aim to address the question of the intercellular response at the transcriptional level within different types of hippocampal neurons from distinct regions after the passive and active transfer of NMDAR autoantibodies. The data showed that excitatory neurons from the DG region exhibited the most dramatically changed expression profile. Genes related to ATP metabolic and biosynthetic processes were up‐regulated in the active model. Differential expression of *Meg3* in interneurons and *Kcnq1ot1* in inhibitory neurons was also observed in different immunization models. These findings may uncover novel therapeutic targets and guide future work for anti‐NMDAR encephalitis.

## 2 METHODS

### 2.1 Mice

C57BL/6 mice (8–10 weeks old, female) weighed about 16–20 g were purchased from the experimental animal center of Southern Medical University (Guangzhou, Guangdong, China). Mice were raised in the laboratory of the experimental animal center of Southern Medical University (Guangzhou, Guangdong, China) under specific pathogen‐free (SPF) conditions. The relative temperature of the feeding environment fluctuated at 25 ± 2°C (RT), the ventilation was good, and the light period (8:00–20:00) of alternating light and shadow was set (8:00–20:00). Mice are free to eat water and food. The follow‐up experiment can be carried out 5–7 days after the mice adapt to the environment in the animal room. Animal experiments were conducted following the ARRIVE guidelines [17]. All animal experiments were approved by the experimental animal ethics committee of Southern Medical University. Animal experiments were carried out blindly, and the experimenters were unaware of the treatment conditions. The collection of clinical samples was approved by the Ethics Committee of The Affiliated Brain Hospital of Guangzhou Medical University and all subjects provided informed consent.

### 2.2 The purification of the anti‐NMDAR encephalitis patient‐derived antibody

The cerebrospinal fluid (CSF) from patients with high titers of anti‐GluN1 ABs (>1:300) during routine clinical examinations were collected. All patients met the clinical diagnostic criteria for anti‐NMDAR encephalitis revised in 2016 [18]. The study protocol was approved by the ethics committee of Nanfang Hospital of Southern Medical University, and the written informed consent of each participant or their guardian was obtained.

Cerebrospinal fluid ABs from patients were isolated and purified using a MelonTM Gel IgG Spin Purification Kit (Thermo Fisher Scientific, Waltham, Massachusetts, USA, #45206). The IgG concentration of purified antibody was determined by using an ND‐1000 ultramicro spectrophotometer (Thermo Fisher Scientific, Waltham, MA, USA). The final concentration was 0.3 mg/ml and stored at –80°C until use.

### 2.3 The induction of the active immunization NMDAR mouse model

C57BL/6 mice (10 weeks old, female) were immunized with GluN1_356–385_ extracellular peptides, which were emulsified in complete Freund's adjuvant (CFA) and supplemented with mycobacterium tuberculosis H37Ra (4 mg/ml), and the final peptide concentration was 1 mg/ml. Mice were injected subcutaneously on the back with 200 μg of peptide in the emulsion mixture and received two enhanced injections with peptide emulsion at 4 and 8 weeks after the first immunization. Mice in the control group received an emulsion mixture of CFA and an equal volume of phosphate‐buffered saline (PBS). All mice were intraperitoneally injected with 200 ng pertussis toxin (List Biological Laboratories) on the day of the last immunization and 48 h later. 12 weeks after the initial immunization, a series of behavioral tests were carried out on mice. The behavior test was conducted at the same time, from 09:00 to 12:00, and the researchers were unaware of the group assignment. Then the mice were sacrificed and the hippocampus was taken for single‐nucleus RNA sequencing.

### 2.4 The induction of the passive immunization NMDAR mouse model

The purified NMDAR patient antibody was diluted to the working concentration of 2 μg/ml. Inject the antibody into the osmotic pump with a syringe with a volume of 200 μl each pump, fill the tubing of appropriate length with 0.9% sterile saline, and assemble the osmotic pump (Alzet, DE, USA, # 2002), flow regulator and tubing (Alzet, DE, USA, #0008851). Place the fully assembled pump into the sterile saline for priming. Place it in a clean 37°C incubator until surgery.

The mice were anesthetized and fixed on the stereotaxic apparatus (RWD Life Science, Shenzhen, China). Make a longitudinal incision in the head and neck of the mouse, insert the pump under the skin of the neck and push it to the left hind limb as far as possible. Fix the catheter tip on the stereotactic instrument, with the bregma as the zero point. Raise the catheter and move 1.0 mm laterally to the right and 0.45 mm posterior, mark and drill here. The original length of the catheter tip is 3.0 mm, after adding a 0.5 mm thick gasket, it moves down vertically at the drilling hole to keep the depth of the catheter tip at 2.5 mm. Glue the catheter and sew the skin. After 2 weeks, a series of behavioral tests were carried out on mice, and then the hippocampus was taken for single‐nucleus RNA sequencing.

### 2.5 Immunofluorescence staining

After PBS and 4% paraformaldehyde were perfused successively through the hearts of mice, the brains were carefully removed and fixed in 4% paraformaldehyde overnight at 4°C. Then, the brain samples were successively dehydrated in 10%–20%–30% sucrose solution and embedded with OCT to obtain a 20‐μm‐thick axial slice. After penetrating and sealing brain slices with 0.3% Triton X‐100 and 10% goat serum respectively, the slices were incubated at 4°C overnight with the primary antibody against IgG. The next day, we incubated the brain slice with a secondary antibody conjugated with DyLight 488 (Abkkine, Beijing, China, #A23220). Finally, the slices were sealed by the Fluoroshield Mounting Medium with DAPI (Abcam, Cambridge, UK, #ab104139) and observed by confocal microscope (Nikon ECLIPSE Ti, Chiyoda Ward, Tokyo, Japan).

### 2.6 The extraction of cerebrospinal fluid

After anesthetizing the mice, a longitudinal incision (about 1 cm) was made along the posterior midline of the neck to strip the surface muscle and the deep muscle attached to the foramen magnum. After exposing the dura mater, gently screw the prepared micropipette. Once the clear CSF is seen rising slowly along the pipette, immediately fix the position of the pipette until the liquid level stops rising and then gently withdraw. Transfer the CSF into a centrifuge tube, and centrifuge at 3000 rpm for 20 min. The supernatant was taken for the concentration of IgG.

### 2.7 Shuttle box active escape experiment

Set the current intensity to 0.5 mA. Put the mice into either side of the box, close the middle door of the box, and make it adapt to the test environment in the box for 5 min. Then start modeling. Conditional stimulation (light or sound, at this time the shuttle door is opened to avoid plantar electrical stimulation) for 3–5 s, followed by non‐conditional stimulation (plantar electrical stimulation) for 15–30 s. If the mice do not cross or have crossed, the middle door shall be closed. Then the animals were given 5–10 s to rest, and the last experiment process was repeated 30 times.

### 2.8 Elevated plus‐maze test

After the mice adapted to the environment, they were placed in the central area of the maze, with heads facing the open arms. Each mouse was placed in the same position. The camera monitor was turned on to record the entry times of open and closed arms and the time of entering each arm within 5 min. After recording, the maze was cleaned and wiped with 75% ethanol to eliminate the influence of odor on subsequent experiments.

### 2.9 Open‐field test

Put the mice into squares facing the wall and let them explore the environment freely for 5 min. The total walking distance, standing times (legs off the ground), walking distance in the middle area, and residence time in the middle area were recorded. Each mouse was placed in the same position. After the test of each mouse, the site was washed with 75% ethanol.

### 2.10 Three‐chamber test

- The first stage: Separate the three boxes with transparent plates, and put the subject mice into the middle box for 5 min. Put stranger 1 randomly into the metal cage in the left or right box, while the metal cage in the other box is empty. Remove the plate so that the subject mice could move freely in three boxes for 10 min. Record the times and duration of direct contact between subject mice and stranger 1 or an empty metal cage (3–5 cm around the metal cage is defined as the contact range). The number and duration of subject mice entering each box (when the mouse's head and four paws enter a box, it is considered to be in that box).
- The second stage: Put the second strange mouse (stranger 2) in the hollow metal cage of the first stage experiment, and then record for 10 min to observe the duration and times of contact between subject mice and stranger 1, stranger 2.

### 2.11 Novel object recognition

Place two objects A and B on the left and right ends of the side wall of the test box and put the mice into the field with their backs facing the two objects. The distance between the mice and the two objects is the same. Record the contact between the mice and objects A and B within 10 min. The test was conducted again 24 h later. At this time, object B was replaced by a new object C. After the mice were placed in the field, the mouse's exploration of object C was observed within 5 min, including the times the mouse's nose or mouth touched the object and the duration within 2–3 cm from the object (the front paw placed on the object, nose smelled the object, licked the object, etc. are defined as exploring the objects).

### 2.12 Forced swimming test

The mice were placed in a plastic cylinder containing warm water (27–28°C), the cylinder is deep enough to prevent the mice from contacting with the bottom and force the mice to swim. The test lasts for 6 min, and the total time of not moving for 4 min is recorded from the second minute. Analyze the static time (the floating state is defined as when the limbs no longer paddle, and only using the small movements of the limbs, head, and tail to maintain balance). Change the water after each test.

### 2.13 Marble burying test

Lay a 5 cm‐thick bedding at the bottom of a 30 cm × 20 cm × 20 cm dry and clean box, and place 12 glass beads (diameter 14–15 mm) evenly on the mat in 3 columns and 4 rows. Put the mice gently in the same corner of the box and observe for 30 min. If more than two‐thirds of the beads were covered with a pad, it is considered that the bead is buried by the mice.

### 2.14 Nest building test

Before the experiment, the mice should be raised separately to adapt to the independent environment in advance. Lace one Nestlet in each cage and supply exactly 3.0 g of Nestlet material per cage. Assess the nests the next morning by weighing untorn nestlet pieces. The definition of an untorn piece is more than 0.1 g. Record the start of the experiment by photographing it. After 20 h, record the present status.

### 2.15 Single‐nucleus library preparation and sequencing

The single‐cell suspensions were used for snRNA‐seq library construction with the Single Cell 3′ Reagent Kit v3.1 (10× Genomics) according to the manufacturer's instructions. The constructed libraries were sequenced on the Illumina HiseqXTEN platform.

### 2.16 Reads alignment and UMI counting

Cell Ranger Software Suite (v6.1.1) was used to perform sample de‐multiplexing, barcode processing, and single‐cell 3′ UMI counting, and STAR was used in alignment with mice mm10 as the reference genome.

### 2.17 Quality control

Gene‐barcode matrices for each sample generated by Cell Ranger were loaded into R as Seurat (v4.0.3) object for filtering, data normalization, dimension reduction, clustering, and gene differential expression analysis. Genes in fewer than three cells were excluded, and the cells with gene numbers between 200 and 4000, UMI count between 500 and 12,500, and mitochondrial gene percentage below 10%.

### 2.18 Data normalization and integration

*SCTransform* was used to normalize each sample and then used to integrate six samples together. Specially, *PrepSCTIntegration* function was run to identify anchors, and the normalization. Method was set to SCT when running *FindIntegrationAnchors* and *IntegrateData* to get the integrated data.

### 2.19 Dimensionality reduction and clustering

The integrated gene‐barcode matrix was used to construct a shared nearest neighbor graph based on the Euclidean distance by the selected significant principal components (dims = 1:40). Cells were clustered at an appropriate resolution and then visualized using a 2‐dimensional t‐distributed Stochastic Neighbor Embedding (t‐SNE) algorithm.

### 2.20 Differential genes expression analysis

Differentially expressed genes (DEGs) for each cell type between the different groups were identified by the function *FindAllMarkers* in Seurat with the following criterion: |LogFC| > 0.25, *p‐*value adjusted by Bonferroni <0.05, minimal percentage in each group >0.25. Visualization of markers was performed by heatmap or dotplot using R packages pheatmap (v1.0.12) or Seurat.

### 2.21 Gene functional enrichment analysis

For DEGs, gene ontology (GO) analysis was performed with R package cluster Profiler (v4.0.2) [19]. Results with *p* value adjusted by Benjamini & Hochberg (BH) < 0.05 were further visualized with the R package ggplot2 (v3.3.5).

### 2.22 Cell trajectory analysis

Cell trajectory for specific cell types was performed using R package monocle (v2.20.0) [20]. DEGs in different groups of each subtype were input as selected genes. Then dimensionality reduction was applied to the data using the algorithm known as Reversed Graph Embedding. Finally, cell ordering was performed using manifold learning by the function *orderCells* and visualized by the function *plot_cell_trajectory*. Besides, branches that appeared in the trajectory were analyzed using branched expression analysis modeling (BEAM) to discover DEGs between the branches by the function *BEAM* and visualized by the function *plot_genes_branched_heatmap*.

### 2.23 Quantitative real‐time PCR (qPCR)

The hippocampus of mice in each group was taken, and after adding Trizol, ultrasound homogenization was carried out. Total RNA was extracted and reverse transcribed to synthesize cDNA. qPCR was used to detect the difference of ATP metabolic and biosynthetic regulators' expression among groups.

### 2.24 Cell culture

The HT‐22 cell line was cultured in DMEM containing 10% FBS. Cells were cultured at 37°C in a humidified environment containing 5% CO_2_ and 95% air.

### 2.25 ATP analysis

The HT‐22 cell line was treated with purified NMDAR patients' antibody (20 μg/ml) for 12 h and then lysed in a lysis buffer for 10 min and centrifuged at 12,000× *g* for 5 min. The supernatant was collected for subsequent measurement. The ATP in the supernatant was determined with an ATP Assay kit (Beyotime) by a microplate reader (Synergy HT; BIOTEK, Broadview, IL). The protein level of the supernatant was determined by the BCA protein assay kit following the manufacturer's instructions. The relative ATP level = ATP value/protein value.

### 2.26 Mitochondrial membrane potential measurement

The HT‐22 cell line was cultured in confocal dishes overnight. Then, the cells were treated with purified NMDAR patients' antibody (20 μg/ml) for 12 h. The cells were washed with PBS and the tetramethylrhodamine ethyl ester perchlorate (TMRE; 50 nM) dye was added to the confocal dishes, and incubated for 30 min in the dark. Eventually, the confocal dishes were placed in an inverted confocal microscope (Nikon ECLIPSE Ti, Chiyoda Ward, Tokyo, Japan) for imaging.

### 2.27 Statistical analysis

All data were analyzed by SPSS version 22.0 (IBM Corp, Armonk, NY, USA), and the histogram was drawn by GraphPad Prism 8.3.0 (GraphPad Software, La Jolla, CA, USA). Data were expressed as mean ± standard deviation (SD). The validation of the behavior test, the detection of IgG concentration, and the ATP level are analyzed by *t*‐test. Except for the above data, all other data are analyzed by One‐way analysis of variance (ANOVA) followed by Tukey's post hoc test. *p*‐value <0.05 was viewed as statistically significant (*p < 0.05; **p < 0.01; ***p < 0.001).

## 3 RESULTS

### 3.1 Behavioral changes of the passive immunization NMDAR mouse model

**FIGURE 1:**
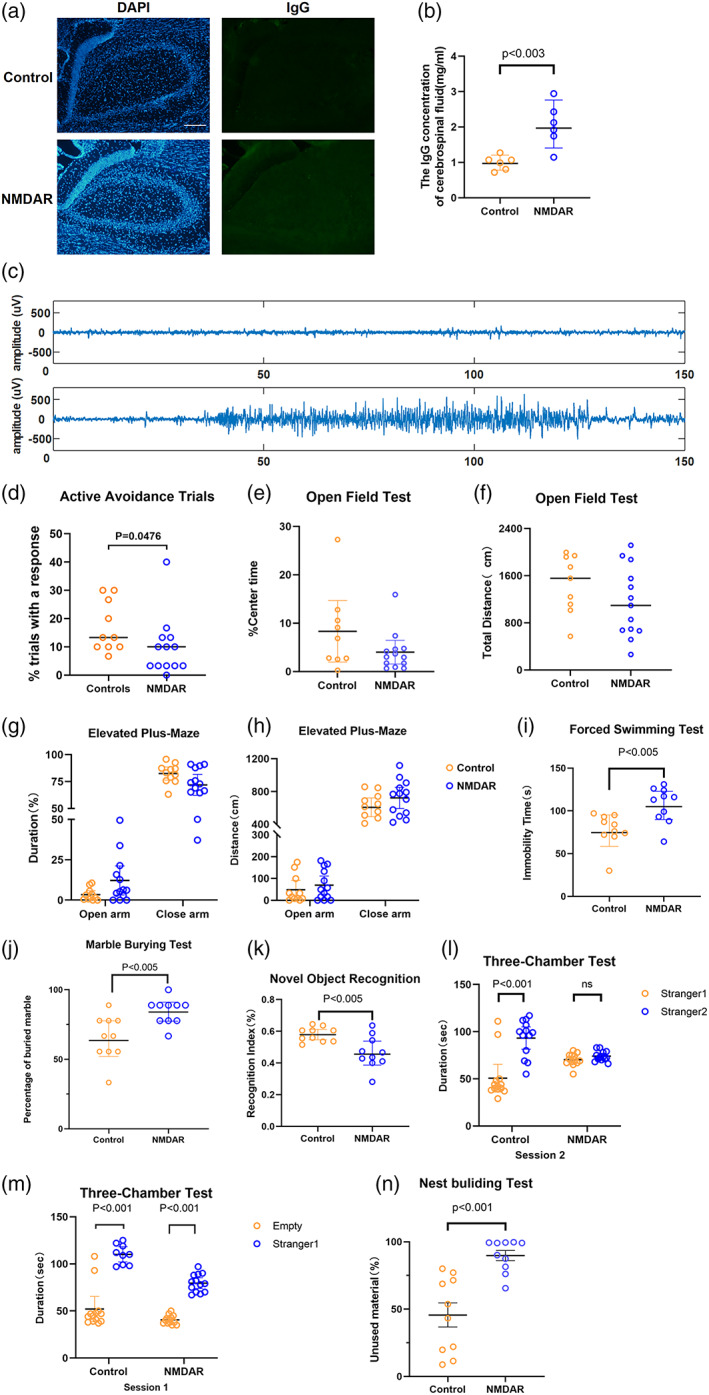
Behavioral changes of mice 2 weeks after injected with N‐methyl‐D‐aspartate receptor (NMDAR) patient‐derived antibody. (A) Immunostaining of IgG in brain slices of mice in the control group and passive immunization NMDAR model group (bar = 200 μm). (B) The concentration of IgG in the cerebrospinal fluid of the mice in the control group and passive immunization NMDAR model group. (C) The EEG data of mice in the passive immunization NMDAR model group (top panel: inter‐ictal period; bottom panel: ictal period). (D) Proportion of active avoidance times of the control group (*n =* 10) and passive immunization NMDAR mouse model (*n =* 13). (E) The control group (*n =* 9) and passive immunization NMDAR mouse model (*n =* 13) were assessed in the open field for time spent in the center area during a 5 min trial. (F) The control group (*n =* 9) and passive immunization NMDAR mouse model (*n =* 13) were assessed in the open field for the total distance during a 5 min trial. (G) The control group (*n =* 11) and passive immunization NMDAR mouse model (*n =* 13) were assessed in the elevated plus‐maze for time spent in the open arms (left) and closed arms (right) during a 5 min trial. (H) The control group (*n =* 11) and passive immunization NMDAR mouse model (*n =* 13) were assessed in the elevated plus‐maze for the total distance in the open arms (left) and closed arms (right) during a 5 min trial. (I) The control group (*n =* 10) and passive immunization NMDAR mouse model (*n =* 10) were assessed in the forced swimming test for the immobility time in the water. (J) The control group (*n =* 10) and passive immunization NMDAR mouse model (*n =* 10) were assessed for the percentage of buried marble. (K) The control group (*n =* 10) and passive immunization NMDAR mouse model (*n =* 10) were assessed for the discrimination index. (L) Session 1 of the three‐chamber test of the control group (*n =* 11) and passive immunization NMDAR mouse model (*n =* 13). (M) Session 2 of the three‐chamber test of the control group (*n =* 11) and passive immunization NMDAR mouse model (*n =* 13). (N) The Proportion of unused material of the control group (*n =* 10) and passive immunization NMDAR mouse model (*n =* 10). (O) Results are expressed as the mean ± SD, *p < 0.05, **p < 0.01, ***p < 0.001 versus the control group.

Schizophrenia‐like symptoms and memory deficits are the main clinical manifestations of anti‐NMDAR encephalitis patients [9]. In our previous study, we found that active immunization NMDAR model mice had shorter exploration time and lower discrimination ability for new objects in the novel object recognition test (NORT); In the three‐chamber test, the exploration time of active model mice to unfamiliar mice was shortened, showing similar memory and cognitive impairment to NMDAR patients [7]. In order to verify the effect of the passive immunization NMDAR model established by pumping purified antibodies from NMDAR patients into the lateral ventricle for 2 weeks on neural function and behavior, we assessed the IgG concentration and conducted a series of behavioral experiments related to cognition, memory, depression, and anxiety. First, we detected the IgG concentration in the hippocampus and CSF of mice, the results showed that the IgG content in the passive immunization NMDAR model group was significantly increased **(**Figure 1A,B **)**. Second, the EEG data was collected according to previously described methods [21], showing that the amplitude of brain waves in the model group mice during the ictal period was significantly higher than the normal value (Figure 1C). These results indicated that we have successfully established the passive immunization model. Furthermore, The behavioral results showed that after 2 weeks of antibody pumping, the proportion of active avoidance trials in the model group was significantly lower than that in the control group (Figure 1D, *p* < 0.05), suggesting that the memory function of the passive immunization model mice was damaged. Similar to the active immunization model, there was no difference in the open‐field test and elevated‐maze test between the two groups (Figure 1E–H). However, we found that in the forced swimming test, the immobility time of mice in the passive immunization model group was significantly prolonged, indicating that the autoantibody derived from NMDAR patients could induce depressive behavior (Figure 1I, *p* < 0.01). In the marble burying test, the number of glass beads embedded by the passive immunization model group was significantly more, which also reflected the increased anxiety level of mice (Figure 1J, *p* < 0.01). In addition, we observed that in the new object recognition test, the exploration tendency of the model group mice was significantly reduced compared with the control group (Figure 1K, *p* < 0.01), and in the three‐chamber test, the control group showed a tendency to explore unfamiliar mice, while the passive NMDAR model mice significantly reduced the number of explorations to unfamiliar mice (Figure 1L,M, *p* < 0.001). In the nest building test, the model group mice left more unused material for the nest, indicating that the passive immunization model group have more severe impairment in social behavior and memory ability (Figure 1N, *p* < 0.001). Nest‐building test, three‐chamber test, and new object recognition test are often used to evaluate social‐spatial memory levels. The results of the above three behavioral experiments suggest that the passive model shows obvious social behavior disorder, which is a typical symptom of schizophrenia. Therefore, the NMDAR model mice immunized passively showed similar clinical symptoms to the NMDAR patients related to memory and schizophrenia. Combined with the active immune model successfully constructed in the early stage [7], we will use these two immune models in further research to explore the pathological mechanism of anti‐NMDAR encephalitis.

### 3.2 Single‐nucleus transcriptome profiling identified different neuron types in mice hippocampus

**FIGURE 2:**
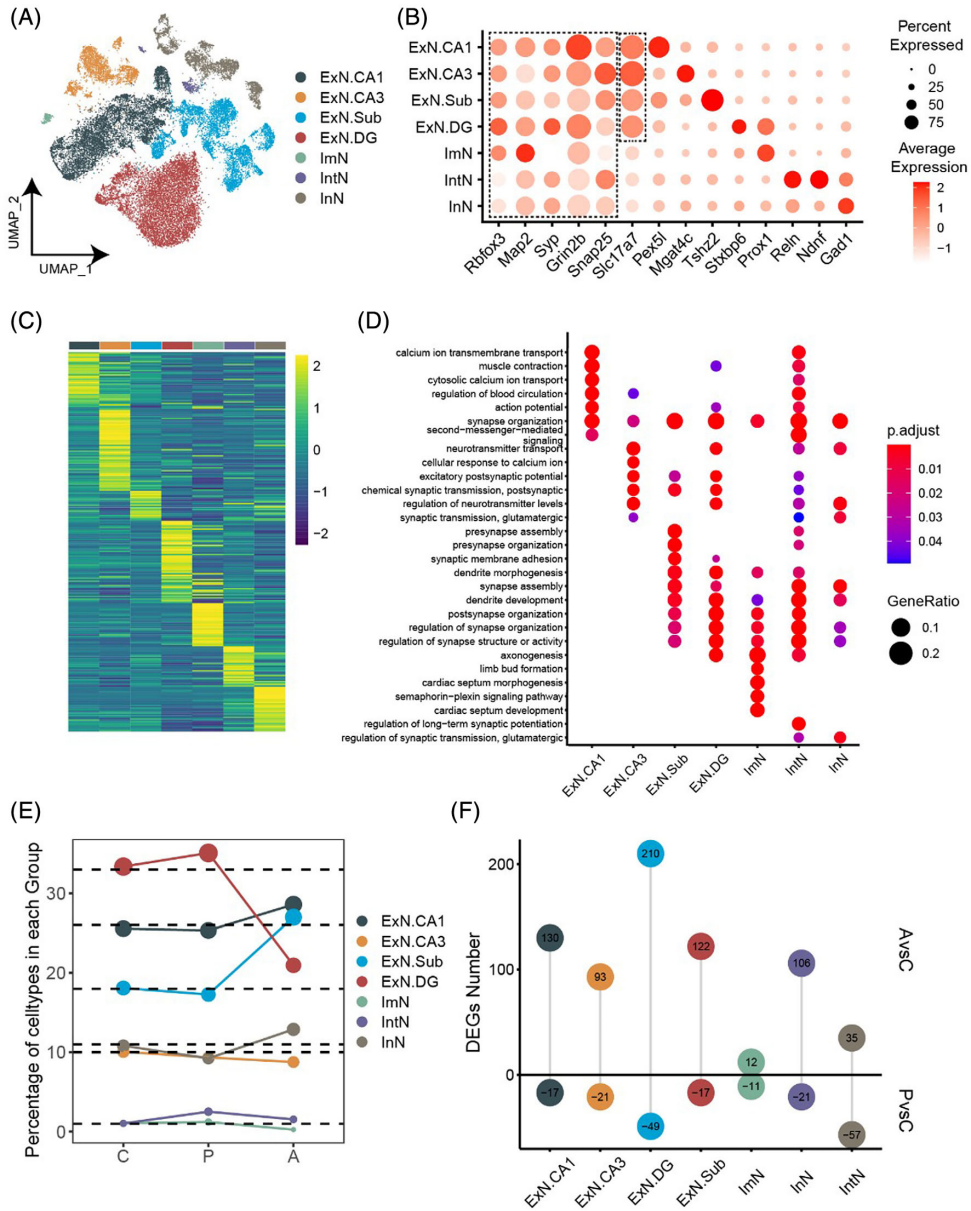
Cell diversity in mice hippocampus neurons delineated by single‐nucleus transcriptome. (A) T‐distributed stochastic neighbor embedding (t‐SNE) plot of 7 neuron cell types. Each dot represents a single nucleus. (B) Dot plots showing the expression levels of representative genes for each cell type. (C) Heatmap showing the expression of differentially expressed genes (DEGs) in each cell type. Each column represents one cell type and each row indicates the expression of one gene. (D) Dot plot showing the top gene ontology terms in different cell types. Cell size indicates gene ratio. The color keys from blue to red indicate the range of adjusted *p*‐value. (E) Line chart showing the percentage of each cell type in different groups. The dashed lines indicate the percentage in the control group. (F) Lollipop chart showing the numbers of DEGs between AvsC (top) as well as PvsC (bottom).

We investigated the dynamic changes in different mice models by the single nucleus transcriptional profiling of the hippocampus from the two control, two passive, and two active mice using 10× Genomics technology. After data quality control at gene and cell levels, a total of 41,013 neurons were kept for further analysis. We identified seven major neuron types (Figure 2A) based on the expressions of canonical markers (Figure 2B) in the mouse hippocampus (expressing *Rbfox3*, *Map2*, *Syp*, *Grin2b*, and *Snap25*) [22, 23, 24, 25], including immature (ImN, expressing *Prox1*), intermediary (IntN, expressing *Reln* and *Ndnf*), inhibitory (InN, expressing *Gad1*) and four excitatory (expressing *Slc17a1*) neurons, therein, ExN.CA1 expressing *Pex5l* located in CA1 region, ExN.CA3 expressing *Mgat4c* located in CA3 region, ExN.Sub expressing *Tshz2* located in Subiculum region and ExN.DG expressing Stxbp6 located in the DG region. The expression profiles of different neuron types showed distinct differences (Figure 2C). Accordingly, function enrichment of DEGs in each cell type (Figure 2D) displayed obvious preference, for instance, ExN. CA1 was associated with calcium ion transmembrane transport, ExN. CA3 could regulate neurontransmitter levels, ExN.Sub and ExN.DG might be associated with synapse assembly and dendrite development. As for ImN, semaphorin‐plexin Signaling pathway was uniquely enriched, and regulation of glutamatergic synaptic transmission was mostly enriched in IntN and InN cells.

We noticed that the percentages of each neuron types varied greatly in different models **(**Figure 2E **)**. Compared with the control group, ExN.CA1, ExN.Sub and InN were slightly decreased in passive and increased in active group, while ExN.DG and ImN showed the opposite trend, ExN.CA3 and IntN showed coincident decreased or increased in two groups, respectively. Similarly, the numbers of DEGs between the active and control groups as well as the passive and control groups also showed distinction in different cell types (Figure 2F). Obviously, ExN.DG presented the most dramatically changed expression profile with 210 up‐ and 49 down‐regulated DEGs.

### 3.3 Cell re‐clustering distinguished subtypes in the major neuron types

**FIGURE 3:**
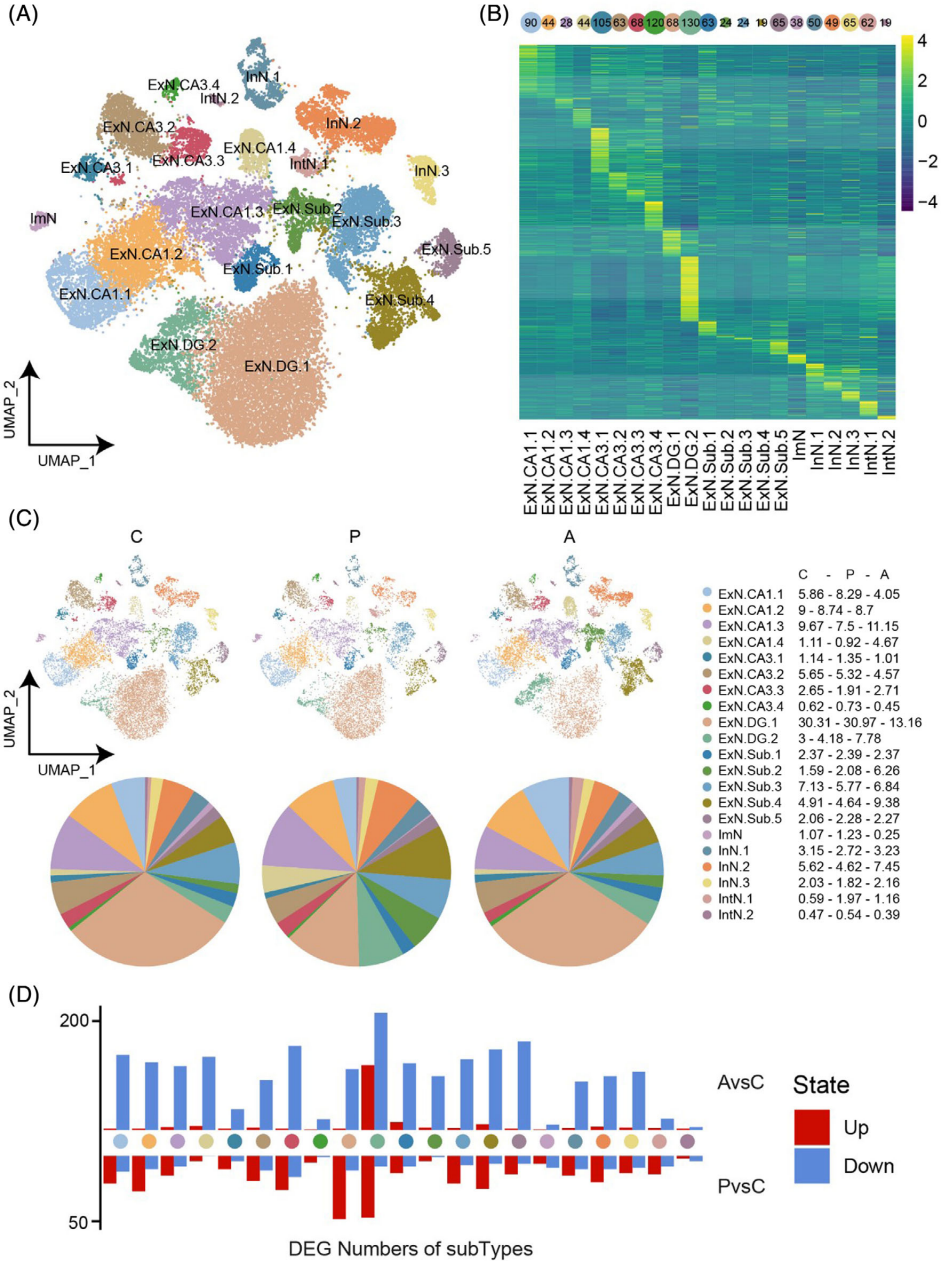
Subtypes identification of the major cell types. (A) TSNE plot of 21 neuron subtypes. Each dot represents a single nucleus. (B) Heatmap showing the differentially expressed gene (DEG) number (top) and expression (bottom) of each subtype. Each column represents one cell type and each row indicates the expression of one gene. (C) TSNE plot of different subtypes in different groups (top). Pie chart showing the percentage of subtypes in each group (bottom). The specific values are listed to the right. (D) Bar plot showing numbers of the up‐and down‐regulated DEGs in each subtype between AvsC as well as PvsC.

Although the major neuron types were identified according to the canonical markers, we observed that there was a contrary tendency in the same cell type, such as ExN.DG (Figure S1). To explore the more detailed cell population in the different model groups, we re‐clustered the above cells and obtained 21 subtypes according to the expression profile and cell proportion (Figure 3A), including four clusters in ExN.CA1, four in ExN.CA3, and two in ExN.DG, five in ExN.Sub, one in ImN, three in InN, two in IntN. DEGs in different subtypes (Figure 3B **)** showed universal (such as ExN.CA1.1 and ExN.CA1.2) or unique (such as ExN.DG.2 and ImN) expression profile, revealing similarities and differences of the gene in the transcriptional expression level. Biological presses shared in the different subtypes (Figure S2), including synapse organization, and regulation of postsynaptic membrane potential. Especially, ExN.DG.2 cells are uniquely enriched in genes related to ATP metabolic process and ribosome biogenesis. More thoroughly, we found that some subtypes increased both in the passive and active groups **(**Figure 3C **)** (including ExN.DG.2, ExN.Sub.1, ExN.Sub.2, ExN.Sub.5, IntN.1), some decreased in the two groups (including ExN.CA1.2, ExN.CA3.2, ExN.Sub.3), some increased in passive while decreased in the active group (including ExN.CA1.1, ExN.CA3.1, ExN.CA3.4, ExN.DG.1, ImN, IntN.2), as well as the opposite (including ExN.CA1.3, ExN.CA1.4, ExN.CA3.3, ExN.Sub.4, InN.1, InN.2, InN.3), suggesting that we should pay attention to the different subtypes. Accordingly, genes exhibited mostly down‐regulated in the active (Figure 3D) compared with the control group, and the up‐regulated genes were mainly located in ExN.DG.2 cells. The number of DEGs in the PvsC comparison was relatively less than in AvsC, in which the up‐regulated genes were more than down‐regulated.

### 3.4 ExN.DG cells exhibit ATP‐related characteristics in the active model

**FIGURE 4:**
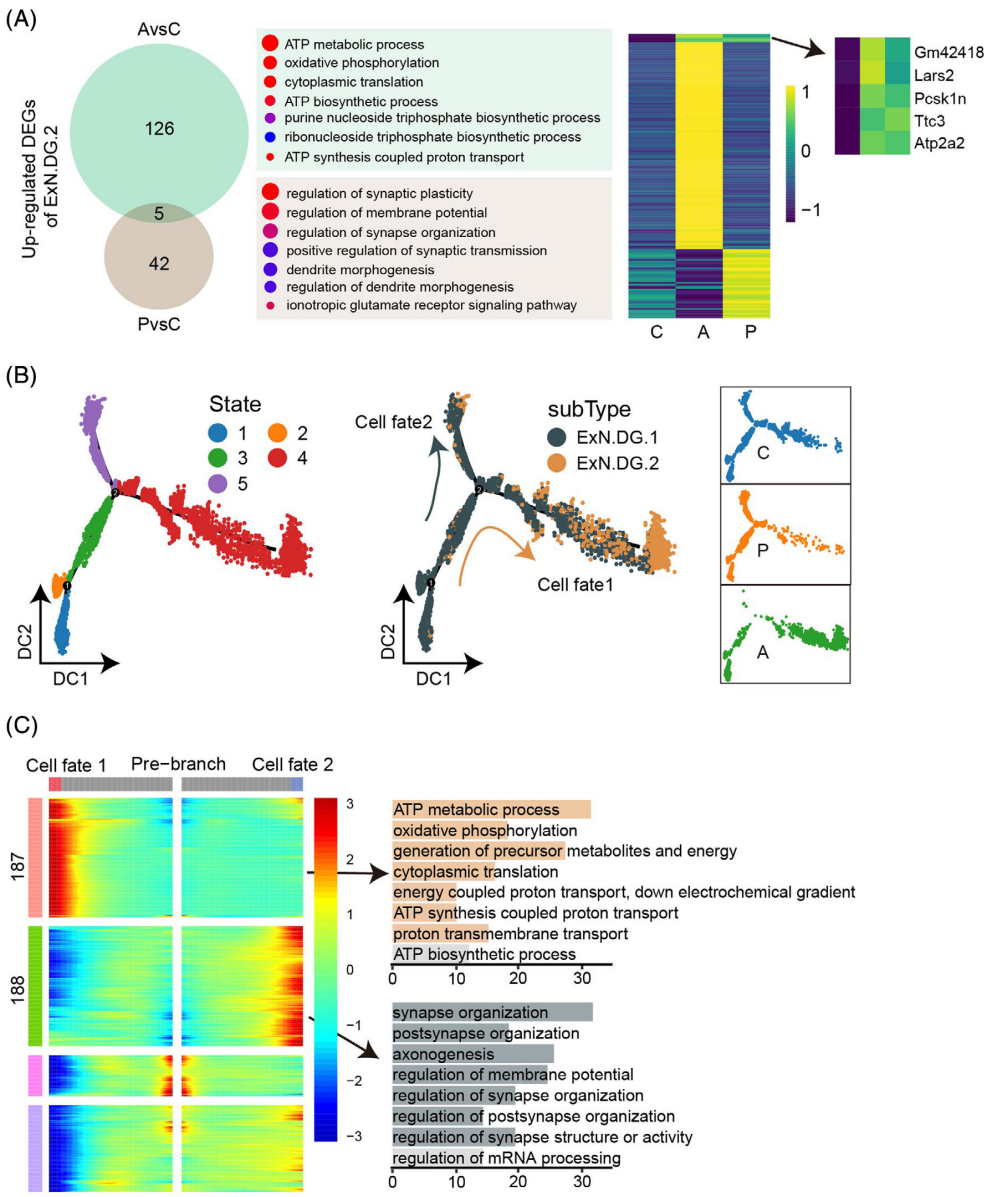
Transcriptional heterogeneity of different mice hippocampus ExN.DG subtypes. (A) Venn diagram (left) displaying the numbers of up‐regulated differentially expressed genes (DEGs) in the active and passive groups compared with the control group. Dot plot (middle) showing the biological processes enriched in the unique DEGs of AvsC (top) and PvsC (bottom) comparison. Heatmap (right) showing the distribution of DEGs in different groups. (B) Pseudotime trajectory indicating the development of ExN.DG neurons and the distribution in different groups. (C) Branched expression analysis modeling showing genes involved in the differential development of Cell fate1 and Cell fate2, and the enriched gene ontology terms were listed at right.

According to the above results, ExN.DG cells contained abundant DEGs in both AvsC and PvsC groups (Figure 3D), and the number of ExN.DG.1 cells decreased while ExN.DG.2 increased in the active group (Figure 3C). Therefore, we further explored the more intense differences between ExN.DG cells. First, the percentages of ExN.DG.2 were increased in the two model groups (Figure 3C), and there were 131 and 47 up‐regulated genes (Figure 4A) in the AvsC and PvsC comparison, respectively, in which *Gm42418*, *Lars2*, *Pcsk1n*, *Ttc3*, and *Atp2a2* were commonly up‐regulated. Unique up‐regulated DEGs in AvsC group were mainly enriched in ATP metabolic and biosynthetic processes, while the unique DEGs in PvsC group were mostly associated with the regulation of synaptic plasticity and dendrite morphogenesis, showing the distinct preference of different models.

Apart from that, we performed cell trajectory analysis by monocle (Figure 4B) to reveal the cellular and molecular dynamic alteration of ExN.DG cells, and explore the reasons for the converse subtypes' proportion changes in the active group. The active group was located at pre‐branch and cell fate1, cell fate2 was enriched by the control and passive groups. Observing the above enrichment, we identified different genes belonging to the different branches during the development of the pseudo‐time trajectory (Figure 4C). Likewise, DEGs enriched in cell fate1, consisting almost entirely of cells from the active group, participated in ATP metabolic and biosynthesis process; DEGs enriched in cell fate2 were associated with synapse organization.

### 3.5 DEGs displayed various trends in IntN and InN cells

**FIGURE 5:**
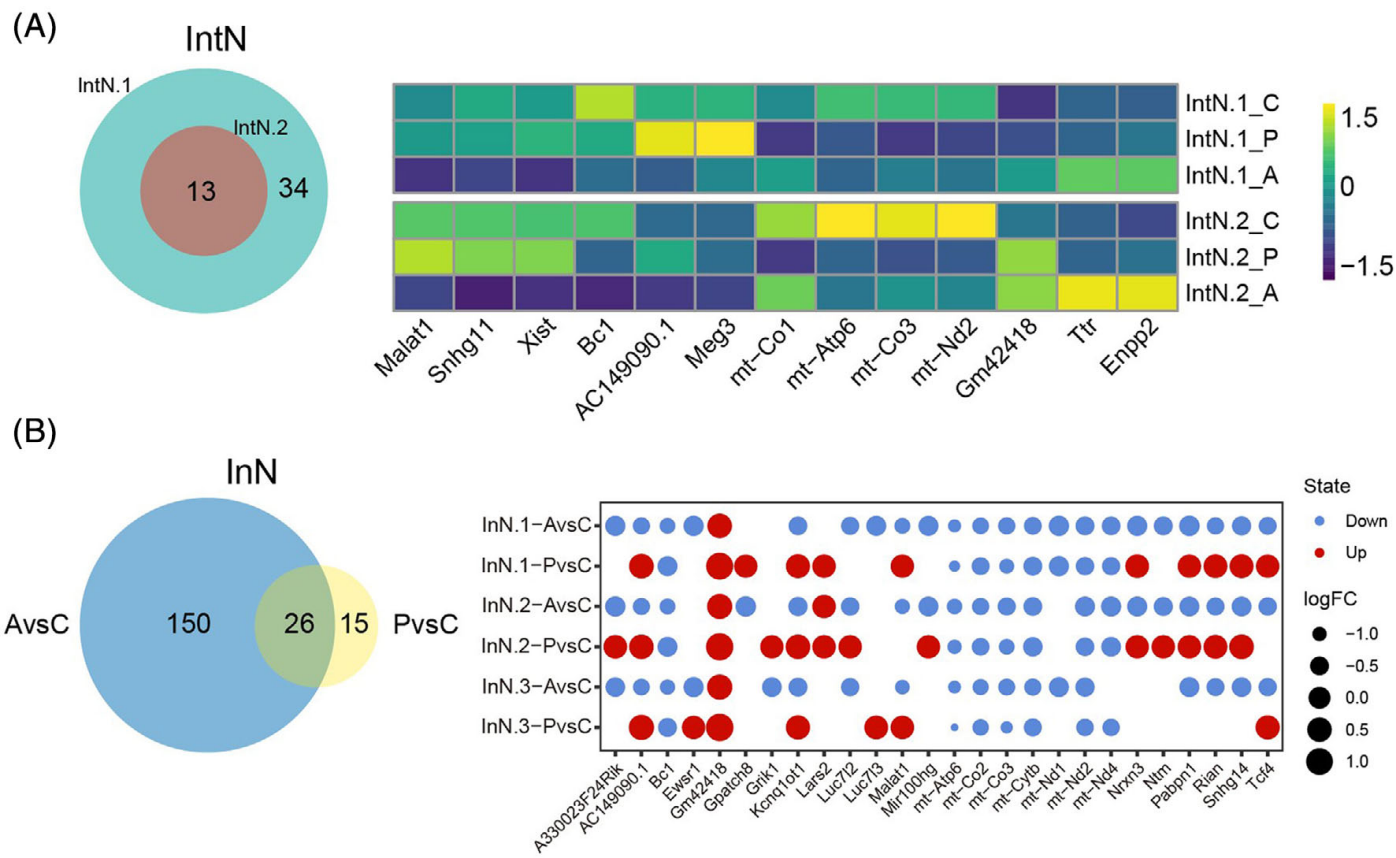
The primary differentially expressed genes (DEGs) in IntN and InN cells among the three groups. (A) Venn diagram (left) displaying the numbers of DEGs in IntN.1 and IntN.2 subtypes. Heatmap (right) showing the distribution of common DEGs in different groups. (B) Venn diagram (left) showing the numbers of DEGs in the active and passive groups compared with the control group of InN cells. Dot plot (right) showing the distribution of common DEGs in different groups.

Two subtypes of IntN cells were increased and decreased in the active group, respectively (Figure 3C), we next filter the curial genes with the reverse fold change state. There were 47 DEGs in the IntN.1 cells, including 13 DEGs co‐regulated in the IntN.2 cells (Figure 5A). The common DEGs were displayed in Figure 5A, it's obvious that most genes showed the same trend in both subtypes, such as *Xist*, *Bc1*, *AC149090.1*, *mt‐Co1*, *mt‐Atp6*, *mt‐Co3*, *mt‐Nd2*, *Gm42418*, *Ttr*, and *Enpp2*. Interestingly, we noticed that *Meg3* was up‐regulated in the IntN.1 while down‐regulated in the IntN.2 cells compared with the control group.

Among the InN cells, the cell proportions of all three subtypes were decreased in the passive while increasing in the active groups, thus we compared the genes that existed in AvsC and PvsC groups (Figure 5B). Undoubtedly, there was a larger difference in the active group which contained 150 unique DEGs apart from 26 common DEGs in the passive group. We could find out that mitochondrial‐related genes, including *mt‐Atp6*, *mt‐Co2*, *mt‐Co3*, *mt‐Cytb*, *mt‐Nd1*, *mt‐Nd2*, *mt‐Nd4*, were down‐regulated in the three subtypes. Besides, several genes showed the opposite changes between AvsC and PvsC comparisons, thereinto, *AC149090.1* and *Kcnq1ot1* were significantly down‐ and up‐regulated in AvsC and PvsC groups, respectively.

### 3.6 Validation of the single‐nucleus RNA sequencing results through in vivo and in vitro models

**FIGURE 6:**
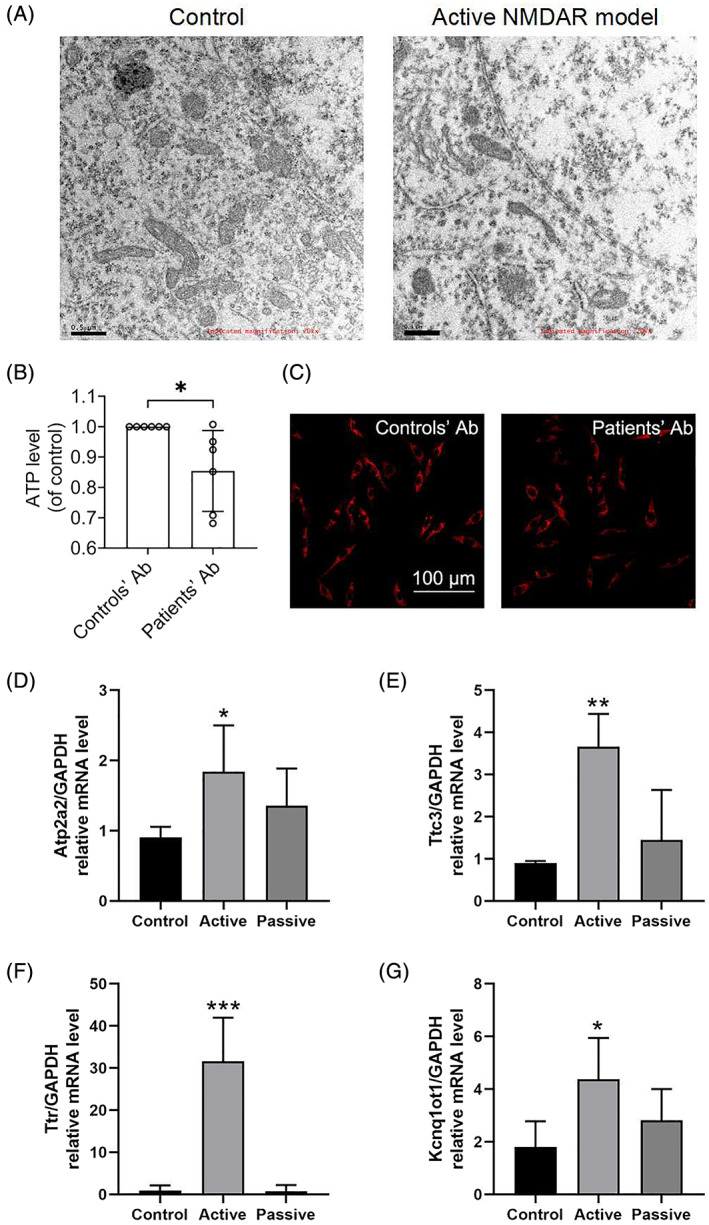
Validation of the single‐nucleus RNA sequencing results in vivo and in vitro models. (A) The mitochondria in the hippocampus of the control group and the active model group were assessed by the electron microscope. (B) The ATP level in HT‐22 cells after stimulation of purified antibody from cerebrospinal fluid in patients of the control group and anti‐N‐methyl‐D‐aspartate receptor (NMDAR) encephalitis group. (C) The mitochondrial membrane potential level in HT‐22 cells after stimulation of purified antibody from cerebrospinal fluid in patients of the control group and anti‐NMDAR encephalitis group. (D) The mRNA level of Atp2a2 in the control group, active immunization group, and passive immunization group. (E) The mRNA level of Ttc3 in the control group, active immunization group, and passive immunization group. (F) The mRNA level of “Ttr” in the control group, active immunization group, and passive immunization group. (G) The mRNA level of Kcnq1ot1 in the control group, active immunization group, and passive immunization group. Results are expressed as the mean ± SD, *p < 0.05, **p < 0.01, ***p < 0.001 versus the control group.

The single‐nucleus RNA sequencing results showed that ExN.DG cells exhibit ATP‐related characteristics in the active model. In order to verify the validity of the sequencing results, the hippocampus of the active immunization model group and the control group were examined by electron microscopy. These results showed that, although no obvious mitochondrial structural damage was seen, the number of mitochondria in the active model group was significantly reduced (Figure 6A). In addition, we stimulated the HT‐22 cell line by purifying the autoantibody of NMDAR patients to establish the in vitro model of NMDAR encephalitis. After stimulation, we could observe a significant decrease in ATP level and mitochondrial membrane potential (MMP) (Figure 6B,C). The above mitochondrial damage‐related results were consistent with the sequencing results.

Next, we detected the changes of ATP metabolic and biosynthetic process‐related genes at the mRNA level, which were up‐regulated in the active immunization NMDAR model. The qPCR data indicated that the mRNA level of *Atp2a2* (Figure 6D, *p* < 0.05), *Ttc3* (Figure 6E, *p* < 0.01), *Ttr* (Figure 6F, *p* < 0.001), and *Kcnq1ot1* (Figure 6G, *p* < 0.05) have also gone up. Therefore, gene changes at the mRNA level can further verify the authenticity of the single‐nucleus RNA sequencing results, although the changing trend of mRNA level between the passive immunization NMDAR model group and the control group was not completely consistent with sequencing results. The minor difference between the sequencing results and mRNA results in the passive immunization model may be due to the detailed analysis of the single‐nucleus RNA sequencing results for various subtypes of different hippocampal neurons, but the samples used for qPCR contain various other types of cells. In conclusion, we have found that the genes with obvious changes in different neuron subtypes mentioned above, such as *Atp2a2, Ttc3, Ttr*, and *Kcnq1ot1* affect the ATP metabolic and biosynthetic process of hippocampal neurons. These genes may be important targets in the pathological process of anti‐NMDAR encephalitis, and it is worth further exploring their potential molecular mechanisms, which may play an important role in the clinical treatment of NMDAR encephalitis in the future.

## 4 DISCUSSION

The active NMDAR immunization model is the disease model established by us through screening a large number of peptide segments, and it is the first time to analyze the single‐nucleus RNA sequencing results of active and passive immunization models of NMDAR encephalitis [7]. Here, we first found that the variation in single‐nucleus profiles of various neurons from different hippocampus regions of passive and active models of anti‐NMDAR encephalitis, which can be leveraged to identify key regulatory modules and the factors involved in the cell fate of hippocampal neurons and the pathophysiological process of anti‐NMDAR encephalitis. In addition, the exhibition of ATP‐related characteristics in different neuronal subtypes was also reported for the first time, so our findings are very innovative and developmental. But we also realized many shortcomings in this research, for example, the outcomes appeared greatly different between the passive and active models. At present, we think that the reason for this discrepancy lies in the different pathological processes induced by the two modeling methods. The active immunization model of anti‐NMDAR encephalitis was induced by subcutaneous injection of GluN1_356–385_ extracellular peptides. Its pathogenesis involves the central nervous system and peripheral inflammatory reaction, accompanied by the activation of complement and the release of a large number of inflammatory factors. In contrast, the passive immunization mouse model formed by pumping autoantibodies from anti‐NMDAR encephalitis patients into the lateral ventricle mainly induces pathological changes through antigen–antibody binding reaction and secondary NMDA receptor invagination, the pathogenic factors of the passive immunization model are relatively simple. Therefore, compared with the passive model, the active immunization model has greater clinical significance and is closer to various pathological manifestations of clinical NMDAR patients, so its results have more reference value for further clinical research.

Patients with hippocampal lesions generally show severe memory deficits, particularly in the episodic [26] and spatial domain [16]. Similarly, mice with experimental anti‐NMDAR encephalitis showed spatial memory impairment [7]. The encoding of spatial memories is segregated through the transverse axis of hippocampal subfields, including the supra‐ and infrapyramidal blades of the DG, and the proximal and distal subregions of CA3 and CA1 [27]. Indeed, DG contains a diverse population of unique cell types [13]. Learning and memory storage are associated with many mechanisms of excitatory and inhibitory synaptic plasticity. Moreover, both excitatory and inhibitory neurons are actively involved in efficient associative memory storage [28, 29]. Specifically, we found that excitatory neurons from CA1, CA3, and DG exhibited a distinct change in DEGs numbers in passive and active models, which might, to some extent, explain the memory deficits in mice with anti‐NMDAR encephalitis. Interestingly, in episodic memory, DG performs pattern separation of cortical inputs before sending its differentiated outputs to CA3, where plastic recurrent excitatory connections facilitate recall of stored patterns in response to partial cues [30, 31]. Indeed, DG receives signals via a major projection from the entorhinal cortex and sends signals to CA3 via the axons of granule cells [32, 33]. Notably, among excitatory neurons from various regions of the hippocampus, we found ExN.DG presented the most dramatically changed expression profile. Hence, the regulation of ExN.DG must be a key therapeutic target for memory deficits of patients with anti‐NMDAR encephalitis.

It is worth noting that ExN.DG exhibited ATP‐related characteristics, which mainly enriched in ATP metabolic and biosynthetic process, in the active model among the dramatically changed expression profile. Undoubtedly, ATP, the main source of chemical energy, plays a fundamental role in mammalian brain function [34]. ATP is consumed predominantly for the maintenance of ion gradients, including Na^+^, K^+^, and Ca^2+^, to ensure that action potentials can be generated and synapses can transmit signals [34]. The reduced Ca^2+^ influx observed in passive model [7] might be contributed to the dramatic change in ATP metabolism in DG neurons. Interestingly, ATP metabolism influences epileptic seizures, another common symptom of anti‐NMDAR encephalitis [1], by the regulation of action potentials [35]. ATP‐sensitive potassium (K_ATP_) channels contribute to slow after hyperpolarization following the burst of action potentials and its open probability is elevated in response to submembrane ATP depletion. Activity‐dependent opening of K_ATP_ channels helps mouse DG neurons act as a seizure gate in the hippocampus and reduces epileptic seizures [35]. Additionally, ATP can enhance LTP in the hippocampus [36]. Factors with the ability to promote aerobic energy metabolism and mitochondrial function physiologically regulate hippocampal synaptic plasticity, cognition, and spatial memory [37]. Recent evidence indicates that ATP‐dependent chromatin remodeling restructures chromatin and such large‐scale changes to chromatin structure further facilitate memory formation [38]. Taken together, factors and modulators involved in ATP metabolic and biosynthetic processes would be the probable factors for further research about the pathophysiological processes of anti‐NMDAR encephalitis.

As aforementioned, inhibitory neurons are also involved in memory storage [28]. *Kcnq1ot1*, a long chromatin‐interacting noncoding RNA that silences ubiquitously imprinted genes in the *Kcnq1* domain via establishing a repressive higher‐order chromatin structure [39, 40], was found significantly changed in hippocampal inhibitory neurons from anti‐NMDAR encephalitis models in this study. Despite the lack of reports about the inner connection among *Kcnq1ot1*, hippocampal inhibitory neurons, and anti‐NMDAR encephalitis models, overexpression of *Kcnq1ot1* is declared to protect hippocampal neurons against anesthesia‐related neural injury [41]. Moreover, *Kcnq1ot1*, regulating target genes expression, contributes to antiepileptic drug resistance [42], which prompts *Kcnq1ot1* as a novel and promising treatment approach for intractable epilepsy. More importantly, *Kcnq1ot1* exhibited the opposite trend in hippocampal inhibitory neurons of different models, it significantly increased in the active model but decreased in the passive model. How this phenomenon arises and what cellular process it regulates during anti‐NMDAR encephalitis are worthy of further exploration.

Interneurons, a minority of the neuronal population, are engaged in the networks exploited for spatial navigation, memory consolidation, and retrieval in the hippocampus [43], in common with excitatory and inhibitory neurons. In contrast to the canonical trisynaptic excitatory circuit, from DG to CA3 to CA1 pyramidal cells, interneurons perform a distinct role in the hippocampal circuit. They primarily provide inhibitory GABAergic synaptic input which transiently hyperpolarizes or shunts the cell membrane away from the action potential threshold [44]. Nevertheless, no report has indicated the role interneurons played in the pathogenic mechanism of anti‐NMDAR encephalitis so far. For the first time, this study implied a differential expression of *Meg3*, a long noncoding RNA, in hippocampal interneurons from mice with anti‐NMDAR encephalitis. Existing evidence have brought to light the association of *Meg3* to hippocampal cognitive recovery [45, 46, 47]. Upregulation of *Meg3* improves spatial learning and memory ability in rats with Alzheimer's disease via inhibiting the pathological injury of hippocampal neurons [45]. *Meg3* also modulates the induction and maintenance of LTP by preserving the level of GluA1 on the plasma membrane of neurons [48]. Surprisingly, *Meg3* dysregulation has been identified in temporal lobe epilepsy through recent RNA sequencing data [49] and *Meg3* overexpression in the hippocampus ameliorates the progression of epilepsy [50]. This literature help assemble a knowledge base of *Meg3* in hippocampal function, but more effort need to be made to characterize the functional implication of *Meg3* in interneurons for cognitive impairment and epileptic seizure of anti‐NMDAR encephalitis.

Taken together, based on single‐nucleus RNA sequencing, this research showed that different types of neurons isolated from the hippocampus at the peak of anti‐NMDAR encephalitis exhibited diverse expression profiles. Several promising candidates have been identified, including ATP metabolic and biosynthetic regulators in excitatory neurons from the DG subregion, *Kcnq1ot1* in inhibitory neurons, and *Meg3* in interneurons. These findings will provide new direction to unveil potent factors for the manipulation of pathophysiological processes and the exploration of etiological therapy for anti‐NMDAR encephalitis.

## CONFLICT OF INTEREST STATEMENT

The authors declare no conflicts of interest.

## Supporting information


**Figure S1.** TSNE plot showing the distribution of different cell types among the three groups.
**Figure S2.** Dot plot showing the enriched biological processes of DEGs in different subtypes.

## Data Availability

The data that support the findings of this study are available from the corresponding author, upon reasonable request. All data were available.
