# Supplementary material for: Single‐nucleus RNA sequencing unveils critical regulators in various hippocampal neurons for anti‐N‐methyl‐D‐aspartate receptor encephalitis
Source: Brain Pathol. 2023 Mar 21;33(4):e13156. doi: 10.1111/bpa.13156 (PMC10307523; doi:10.1111/bpa.13156)
Supplement: Supplementary file 1 — Figure S1. TSNE plot showing the distribution of different cell types among the three groups. Figure S2. Dot plot showing the enriched biological processes of DEGs in different subtypes. [file BPA-33-e13156-s001.docx]

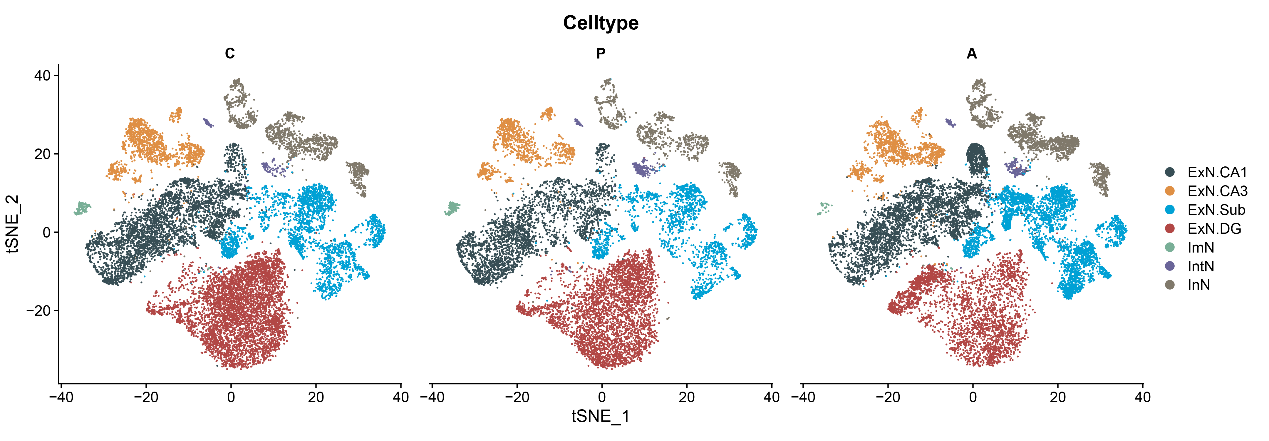


**Figure S1 TSNE plot showing the distribution of different cell types among the three groups.**


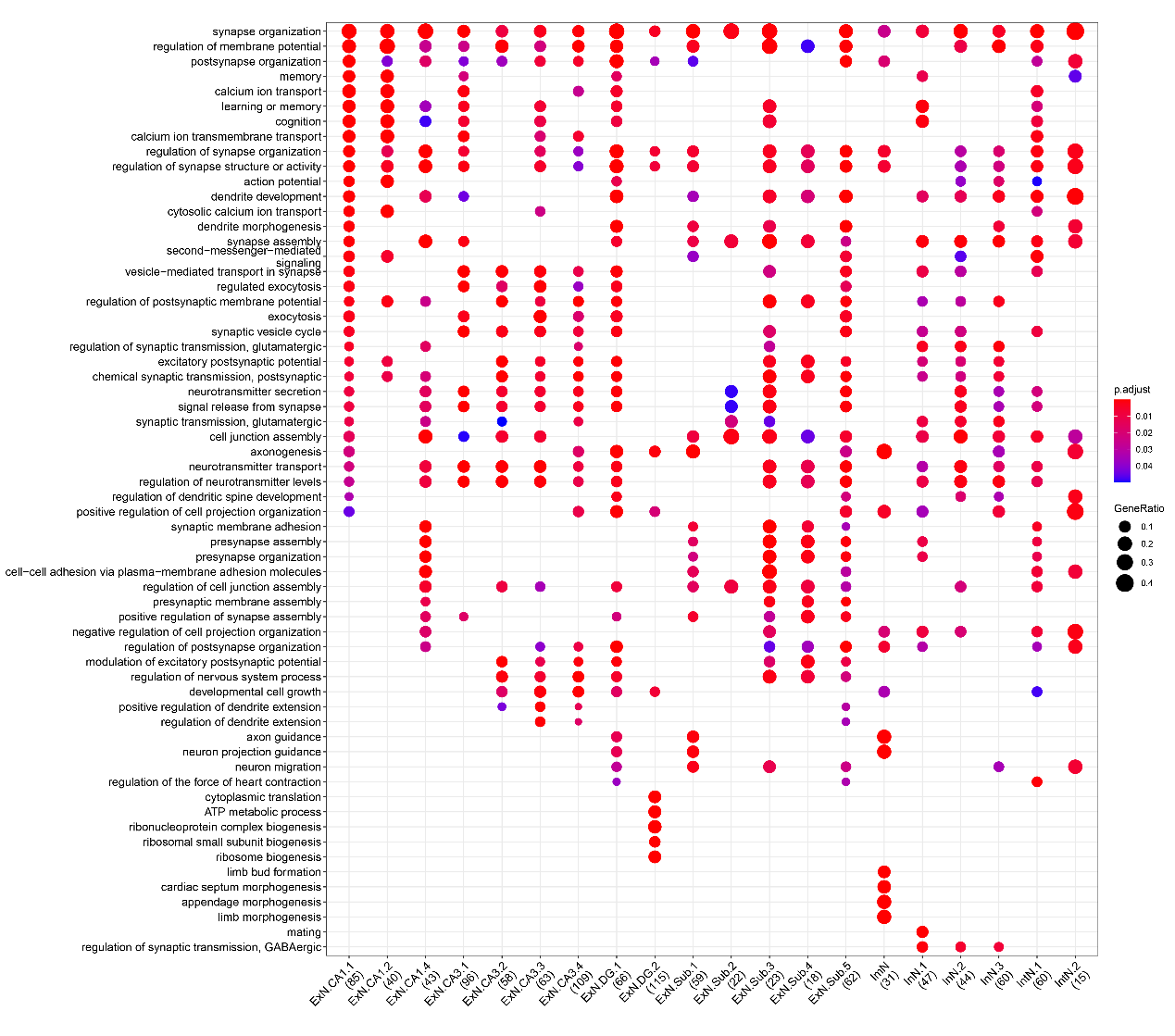


**Figure S2 Dot plot showing the enriched biological processes of DEGs in different subtypes.**
